# AFMNet: A Dual-Domain Collaborative Network with Frequency Prior Guidance for Low-Light Image Enhancement

**DOI:** 10.3390/e27121220

**Published:** 2025-12-01

**Authors:** Qianqian An, Long Ma

**Affiliations:** School of Computer Engineering, Shenyang Jianzhu University, Shenyang 110168, China

**Keywords:** dual-domain network, entropy, frequency prior guidance, fourier transform, information theory, low-light image enhancement

## Abstract

Low-light image enhancement (LLIE) degradation arises from insufficient illumination, reflectance occlusion, and noise coupling, and it manifests in the frequency domain as suppressed amplitudes with relatively stable phases. To address the fact that pure spatial mappings struggle to balance brightness enhancement and detail fidelity, whereas pure frequency-domain processing lacks semantic modeling, we propose AFMNet—a dual-domain collaborative enhancement network guided by an information-theoretic frequency prior. This prior regularizes global illumination, while spatial branches restore local details. First, a Multi-Scale Amplitude Estimator (MSAE) adaptively generates fine-grained amplitude-modulation maps via multi-scale fusion, encouraging higher output entropy through adaptive spectral-energy redistribution. Next, a Dual-Branch Spectral–Spatial Attention (DBSSA) module—comprising a Frequency-Modulated Attention Block (FMAB) and a Scale-Variable Depth Attention Block (SVDAB)—is employed: FMAB injects the modulation map as a frequency-domain prior into the attention mechanism to conditionally modulate the amplitude of value features while keeping the phase unchanged, thereby helping to preserve structural information in the enhanced output; SVDAB uses multi-scale depthwise-separable convolutions with scale attention to produce adaptively enhanced spatial features. Finally, a Spectral-Gated Feed-Forward Network (SGFFN) applies learnable spectral filters to local features for band-wise selective enhancement. This collaborative design achieves a favorable balance between illumination correction and detail preservation, and AFMNet delivers state-of-the-art performance on multiple low-light enhancement benchmarks.

## 1. Introduction

In recent years, low-light image enhancement (LLIE) has become a core task in computer vision, with widespread applications in autonomous driving, surveillance, computational photography, and medical imaging. Images captured under poor lighting conditions often suffer from low contrast, severe noise, color shift, and loss of high-frequency details, which not only degrade perceptual quality but also hinder high-level vision tasks such as object detection and semantic segmentation. This underscores the importance of developing LLIE algorithms capable of restoring illumination, suppressing noise, and preserving structural details.

Traditional LLIE methodologies are typically categorized into histogram-based and Retinex-based approaches. Histogram Equalization (HE) [1] methods enhance contrast by expanding dynamic range but often lack semantic awareness, resulting in overexposure and detail loss. Retinex-based methods [2] model an image as the product of illumination and reflectance, supported by physically interpretable priors. However, these priors—especially the assumption of smooth illumination—struggle in real-world images with complex lighting, leading to suboptimal decomposition and various artifacts such as halos and color distortion.

With the rise of deep learning, convolutional neural network (CNN)-based approaches have become the mainstream in low-light image enhancement (LLIE), delivering notable progress. From Retinex-integrated models such as RetinexNet [3] and KinD [4], to lightweight unsupervised methods like Zero-DCE [5], and more complex designs like RUAS [6], these networks have demonstrated strong capabilities in brightness enhancement.

However, the existing methods suffer from a fundamental limitation: an overwhelming reliance on spatial-domain operations, with no explicit modeling of frequency-domain characteristics. This “spatial-domain bias” leads to several critical issues: (1) limited receptive fields hinder global illumination consistency, often causing uneven brightness; (2) models amplify both noise and fine details due to weak discrimination; and (3) unconstrained spatial processing distorts frequency structures, resulting in artifacts like ringing or aliasing.

While spatial-domain bias limits structural fidelity, another inherent issue lies in information degradation caused by photon scarcity. From an information-theoretic perspective, low-light image acquisition can be regarded as a high-information-loss process: photon scarcity suppresses the Shannon entropy (a measure of information content) of pixel distributions, producing narrow dynamic ranges and degraded spatial variation. This motivates the use of entropy not merely as a metric, but as a guiding principle for restoration. Recent studies confirm this direction—for instance, Wu et al. [7] employ an entropy-based divide-and-conquer strategy, while Kumar et al. [8] introduce an entropy-driven approach aimed at reducing visual defects. Inspired by these works, we propose to internalize entropy awareness into a learnable frequency-domain prior, aiming to enrich spectral energy distribution and recover lost visual structures through dual-domain collaboration.

Building upon these entropy-aware insights, we introduce AFMNet, a novel dual-domain collaborative enhancement framework that decomposes low-light image enhancement into two synergistic tasks: frequency-domain prior estimation and spatial detail restoration. The core innovation lies in a learnable frequency prior guidance mechanism. Specifically, a Multi-Scale Amplitude Estimator (MSAE) is designed to adaptively generate amplitude modulation maps from low-light images. These maps are injected into the Frequency-Modulated Attention Block (FMAB) to guide the frequency-domain attention mechanism, enhancing responses in energy-suppressed regions. Meanwhile, a Scale-Variant Depth Attention Block (SVDAB) leverages multi-scale depthwise separable convolutions and scale-aware attention to adaptively model spatial structural details. Through the collaborative design of frequency and spatial priors, AFMNet achieves a balanced trade-off between illumination enhancement and structural restoration.

The main contributions of this paper are summarized as follows:

- We propose AFMNet, the first framework that decomposes low-light image enhancement into synergistic frequency prior estimation and spatial domain refinement. It learns a physically interpretable amplitude modulation prior to actively guide feature reconstruction, resolving the fundamental trade-off between global illumination consistency and local detail preservation.
- We innovatively design the Multi-Scale Amplitude Estimator (MSAE), which generates pixel-level, content-adaptive amplitude maps to overcome the limitations of static modulation. This module reframes illumination enhancement as a data-driven spectral regression task, enabling scene-specific compensation and significantly improved generalization under non-uniform or multi-source lighting conditions.
- We propose the first attention mechanism that injects a learnable spectral prior into feature modulation for global energy-aware enhancement, and the first feed-forward design that applies learnable complex-valued filters within local patches for structure-preserving, band-selective refinement—together enabling adaptive dual-domain collaboration.
- Extensive experiments on three public benchmarks (LOL-v2-real [9], LOL-v2-synthetic [9], and SDSD-indoor [10]) demonstrate that our proposed AFMNet achieves state-of-the-art performance and generalization, outperforming most competing methods across multiple metrics.

## 2. Related Work

### 2.1. Traditional Methods

Early low-light image enhancement (LLIE) techniques relied on classical image processing algorithms. Histogram-based methods, such as Histogram Equalization (HE) [1] and CLAHE, enhance global contrast by redistributing pixel intensities. Despite their efficiency, they lack content-awareness and often produce artifacts under uneven lighting, including oversaturation and noise amplification. Another major branch is based on Retinex theory [2], which models an image as the product of illumination and reflectance. Methods like SSR and MSR attempt to recover reflectance by estimating and eliminating illumination. However, Retinex-based decomposition is highly ill-posed and depends heavily on handcrafted priors, such as assuming smooth illumination. These assumptions limit robustness in scenes with spatially varying textures and illumination, leading to halos and color distortions.

### 2.2. Deep Learning Methods

Early learning-based LLIE methods were largely driven by CNNs. RetinexNet [3] and KinD [4] combined physical priors with CNNs via a decomposition-enhancement pipeline, and Zero-DCE [5] proposed zero-reference curve learning. Although these networks effectively enhance image brightness, they tend to amplify noise. Similarly, Deep Image Prior (DIP) [11] avoids training data entirely by optimizing a randomly initialized CNN for each image—leveraging network architecture as an implicit prior. While both Zero-DCE and DIP reduce dependency on labeled datasets, they suffer from suboptimal generalization (Zero-DCE) or slow per-image optimization (DIP). SNR-Net [12] introduced an SNR-aware mechanism for adaptive smoothing in noisy regions while preserving structural details. However, CNN-based methods remain fundamentally limited by their local receptive fields, hindering consistent global illumination modeling.

To address this challenge, Transformers [13] have been introduced into LLIE. Hybrid architectures like STAR [14] utilize parallel CNN and Transformer branches for joint local-global feature modeling, while pure Transformer models, such as Uformer [15] and Restormer [16], leverage spatial-domain self-attention to model long-range pixel correlations yet remain fundamentally confined to the spatial domain without explicit frequency-awareness or spectral prior guidance.

More recently, Retinexformer [17] integrates Retinex decomposition into a pure Transformer architecture, using spatial self-attention to model illumination and reflectance in separate branches. While it achieves strong performance by capturing long-range dependencies, it remains confined to the spatial domain—lacking explicit frequency-domain modeling or global spectral priors. As a result, illumination adjustment can be inconsistent in large dark regions, and fine textures may be over-smoothed due to unconstrained spatial attention. In contrast, our AFMNet introduces a frequency-prior-guided attention mechanism, enabling illumination recovery to be driven by learnable spectral energy distributions rather than purely spatial similarity—a conceptual shift that enhances both global consistency and local fidelity.

Generative methods, especially diffusion-based models like AnlightenDiff [18], have also gained attention. These models perform reverse denoising from Gaussian noise to generate enhanced images, offering strong realism and texture restoration. Nonetheless, they often suffer from high computational cost and risk detail loss or oversmoothing when guided by imprecise conditions.

### 2.3. Entropy-Guided and Information-Theoretic Methods

From an information-theoretic perspective, low-light image degradation can be viewed as a process of information loss, where photon scarcity reduces the image’s Shannon entropy (a measure of information content) by compressing its dynamic range and obscuring details. Consequently, recent research has started to leverage entropy not just as a post-hoc evaluation metric, but as an active guiding principle during the enhancement process. For  instance, Wu et al. [7] proposed a lightweight, entropy-based network that adopts a “divide-and-conquer” strategy to handle different image regions. Similarly, Kumar et al. [8] introduced an entropy-driven approach specifically designed to enhance images while reducing visual defects and artifacts. While these methods validate the effectiveness of using entropy awareness, they are often confined to the spatial domain and typically require explicit, computationally intensive entropy map calculations during inference, failing to address the fundamental signal suppression in the frequency domain.

### 2.4. Frequency Domain-Based Methods

Parallel to spatial-domain innovations, other studies have explored tackling LLIE in the frequency domain by directly modulating the Fourier spectrum. FourLLIE [19] demonstrated that brightness can be enhanced via learnable amplitude adjustment. However, its simplistic strategy—applying a global modulation map without content adaptation—and reuse of the original phase often result in color distortion and artifacts. DMFourLLIE [20] improved upon this by jointly enhancing both amplitude and phase, but it relies heavily on infrared priors and incurs high computational overhead.

At a conceptual level, both methods adhere to a compensation-after-learning paradigm: the network first learns spatial features in isolation, and spectral adjustment is applied only as a final corrective step, disconnected from the feature formation process. This creates a fundamental limitation: without frequency-domain awareness during learning, the network cannot adaptively allocate enhancement effort where spectral energy is most deficient.

The importance of phase information is also recognized in phase retrieval tasks. For example, Res-U2Net [21] explicitly reconstructs phase for 3D structure recovery—requiring physical model inversion. In contrast, our FMAB and SSFBlock preserve input phase to maintain structural coherence during amplitude modulation, avoiding the complexity of phase estimation while achieving stable enhancement.

AFMNet challenges this paradigm by introducing a prior-guided co-evolution framework: frequency prior estimation is not an endpoint, but a guiding thread woven throughout the architecture. The estimated spectral prior does not merely scale outputs—it actively shapes how spatial features are attended to, refined, and fused. This represents a shift from reactive compensation to proactive guidance—from isolated post-processing to integrated, theory-driven collaboration between domains.

These approaches, though more aligned with the global nature of illumination, tend to use coarse strategies or depend on auxiliary data, lacking the adaptive, theoretically-grounded spectral control that enables robust performance under diverse lighting conditions.

To bridge the gaps left by existing methods—the limited receptive fields of spatial-only models, the spatial confinement and computational inefficiency of current entropy-guided approaches, and the lack of content-adaptive control in prior frequency-domain techniques—we propose AFMNet. Our framework introduces a novel paradigm by synergizing information-theoretic principles with frequency-domain operations. It features a learnable prior network that adaptively modulates the Fourier amplitude spectrum at the pixel level, guided by a hybrid-domain attention mechanism. This design deeply fuses frequency-domain global enhancement with spatial-domain local refinement, creating an efficient, adaptive, and theoretically grounded solution to the LLIE problem.

## 3. Methodology

**Figure 1:**
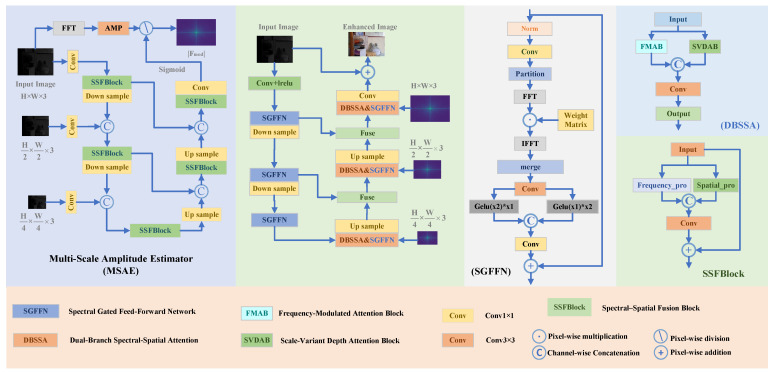
AFMNet overview: MSAE is the amplitude estimation module, used to guide FMAB. SVDAB is the spatial refinement module, designed to enhance local textures and structural details. SGFFN performs learnable frequency-band selective enhancement in the feed-forward path.

This section details our proposed Adaptive Fourier Modulation Network (AFMNet). Framed from an information-theoretic perspective, our methodology aims to solve Low-Light Image Enhancement (LLIE) as an information recovery task. The core idea is to first estimate the information deficit in the frequency domain and then guide a dual-domain network to compensate for this loss, thereby maximizing the information content of the output. We first outline the overall framework (Figure 1), then describe its two core stages: (1) Frequency Prior Guidance for information deficit estimation, and (2) Dual-Domain Detail Refinement for information compensation and restoration. Finally, we present the compound loss function designed to supervise this information recovery process.

### 3.1. Multi-Scale Amplitude Estimator

To bridge the gap between spatial context modeling and frequency-domain enhancement, we introduce the Multi-Scale Amplitude Estimator (MSAE)—a module that, to our knowledge, is the first to explicitly frame low-light restoration as a learnable *frequency prior estimation* task. Trained end-to-end, MSAE adaptively generates pixel-level amplitude modulation maps to selectively amplify energy-suppressed regions in the Fourier spectrum, using residual connections for stable gradient flow.

Unlike spatial-domain methods that lack global illumination awareness, MSAE captures scene-wide spectral priors; unlike conventional frequency-domain approaches that rely on handcrafted or static spectral filters, it learns content-adaptive modulation, greatly improving robustness under non-uniform lighting. Within AFMNet, MSAE serves as the frequency prior generator: it provides explicit guidance to FMAB on where spectral energy should be compensated, preventing blind amplification and visual artifacts such as overexposure or halo effects—as empirically validated by our ablation study (Table 4), where removing MSAE leads to significant performance degradation.

The multi-scale U-Net backbone is essential: low-light degradation exhibits hierarchical patterns—fine textures suffer localized photon loss (requiring high-frequency compensation), while large dark regions need global energy redistribution (low-frequency emphasis). A single-scale estimator cannot simultaneously model both phenomena.

Structurally, MSAE adopts a three-level U-Net backbone with cross-scale fusion, where each processing block is implemented as an SSFBlock (Spectral–Spatial Fusion Block, detailed in Section Spectral–Spatial Fusion Block) to enable dual-domain feature interaction during prior estimation.

The input pyramid includes the original image and its bilinearly downsampled versions at half and quarter resolutions (*x*, $x_{s2}$, $x_{s3}$). Each encoder level integrates features from lower scales to enrich the representation, with the first encoder directly processing the high-resolution input.

(1) $$F_{\mathrm{enc},1}=\mathrm{SSFBlock}\left(x\right).$$

In the encoder, deeper layers (i∈{2,3}) fuse the downsampled feature $F_{\mathrm{enc},i-1}$ from the previous level and the corresponding input image $x_{si}$ as follows:

(2) $$F_{\mathrm{enc},i}=\mathrm{SSFBlock}\left(\mathrm{Concat}\left(\mathrm{Downsample}\left(F_{\mathrm{enc},i-1}\right),{\mathrm{Conv}}_{1\times 1}\left(x_{si}\right)\right)\right).$$

The decoder adopts a symmetric structure, progressively upsampling and combining features via skip connections:

(3) $$F_{\mathrm{dec},i}=\mathrm{SSFBlock}\left(\mathrm{Concat}\left(\mathrm{Upsample}\left(F_{\mathrm{dec},i+1}\right),F_{\mathrm{enc},i}\right)\right).$$

The output of the last decoder level, $F_{\mathrm{dec},1}$, is passed through a 1×1 convolution and a Sigmoid function to generate the amplitude prior map:

(4) $$M_{\mathrm{prior}}=\mathrm{Sigmoid}\left({\mathrm{Conv}}_{1\times 1}\left(F_{\mathrm{dec},1}\right)\right).$$

Finally, the modulation is applied to the Fourier amplitude spectrum of the input image:

(5) $$|F_{\mathrm{mod}}|=\frac{\left|F(x)\right|}{M_{\mathrm{prior}}+\epsilon },$$

where F(·) denotes the Fast Fourier Transform (FFT), and ϵ is a small constant to prevent division by zero. This formulation reframes illumination correction as an entropy-maximization process: regions with severe spectral suppression (low $M_{\mathrm{prior}}$) receive stronger amplification, pushing the output toward higher Shannon entropy. The loss term $L_{\mathrm{freq}}$—measuring cosine similarity between $|F_{\mathrm{mod}}|$ and $|F_{\mathrm{GT}}|$—further regularizes this process, compelling MSAE to learn physically plausible spectral distributions that serve as strong priors for downstream spatial refinement.

#### Spectral–Spatial Fusion Block

The Spectral–Spatial Fusion Block (SSFBlock) is designed to address a core challenge in low-light prior estimation: the need to simultaneously model global illumination structure and local texture fidelity. These two properties are inherently encoded in different domains—illumination patterns manifest as low-frequency spectral distributions, while textures and edges reside in high-frequency spatial gradients. Processing them in isolation leads to incomplete representations: pure spatial models miss global context, while pure spectral models lose fine localization.

SSFBlock resolves this by maintaining parallel, domain-specific processing paths. The frequency branch operates on the Fourier spectrum to capture energy distribution patterns without spatial discretization artifacts. The spatial branch preserves pixel-level structural cues through invertible nonlinear mapping. Only after independent refinement are the two streams fused. This design ensures that neither domain dominates prematurely, allowing MSAE to base its prior estimation on a balanced, complementary feature representation.

Effective feature extraction requires combining fine-grained local details with global context. Convolutional neural networks (CNNs) excel at modeling local structures but struggle with long-range dependencies, whereas spectral representations naturally capture global relations. To bridge this gap, we propose the Spectral–Spatial Fusion Block (SSFBlock; see Figure 1, right), comprising parallel frequency and spatial branches.

**Figure 2:**
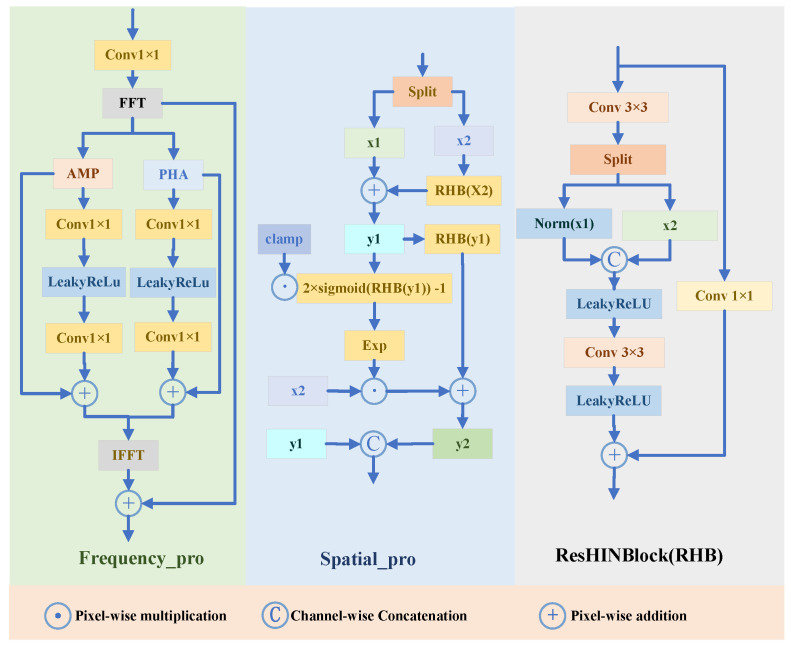
The framework of the proposed SSFBlock.

In the frequency branch (FreBlock; Figure 2, left), an input feature map *x* is first projected in the spatial domain via a 1×1 convolution and then mapped to the Fourier domain using a 2D real FFT (rfft2) to obtain F(x). The spectrum is decomposed into amplitude |F(x)| and phase ∠F(x), which are processed by two explicit sub-branches. Each sub-branch consists of Conv1×1→ LeakyReLU → Conv1×1 and is added back residually to its corresponding component for lightweight, dynamic enhancement. The enhanced spectrum is finally transformed back to the spatial domain via the inverse real FFT (irfft2). The operations are

(6) $$\begin{array}{cc} |F(x)|\; & =\;\mathrm{abs}(rfft2(x)),\\ ∠F(x)\; & =\;\mathrm{angle}(rfft2(x)),\\ |F_{\mathrm{enhanced}}|\; & =\;|F\left(x\right)|+{\mathrm{Conv}}_{1\times 1}\;\left(\mathrm{LeakyReLU}({\mathrm{Conv}}_{1\times 1}\left(|F\left(x\right)|)\right)\right),\\ ∠F_{\mathrm{enhanced}}\; & =\;∠F\left(x\right)+{\mathrm{Conv}}_{1\times 1}\;\left(\mathrm{LeakyReLU}({\mathrm{Conv}}_{1\times 1}\left(∠F\left(x\right)\right))\right). \end{array}$$

The Fourier spectrum is decomposed into amplitude and phase because they govern distinct perceptual dimensions. Amplitude modulates perceived brightness and contrast—regions with suppressed amplitudes correspond to areas where photon counts are low, requiring energy compensation. Phase determines geometric coherence—misalignment in phase disrupts edge continuity and introduces structural artifacts. By refining them through separate sub-networks, SSFBlock enables targeted adjustment: the amplitude branch learns to redistribute spectral energy according to contextual illumination needs, while the phase branch stabilizes structural layout to prevent distortion. This separation is not arbitrary—it aligns with the physical decoupling of luminance and geometry in image formation.

The enhanced amplitude and phase spectra are recombined into a complex spectrum using Euler’s formula: $e^{j\theta }=\cos\left(\theta \right)+j\sin\left(\theta \right)$. The reconstructed spectrum is mapped back to the spatial domain via the inverse real FFT (irfft2), and added to the original input *x* through a residual connection to produce the final output:

(7) $$\begin{array}{cc} F_{\mathrm{recon}}\left(x\right) & =\left|F_{\mathrm{enhanced}}\right|⊙e^{j∠F_{\mathrm{enhanced}}},\\ x_{\mathrm{freq}} & =irfft2\left(F_{\mathrm{recon}}\left(x\right)\right),\\ \mathrm{Fre}\_\mathrm{out} & =x_{\mathrm{freq}}+x, \end{array}$$

where *j* is the imaginary unit and ⊙ denotes the Hadamard product (element-wise multiplication). As illustrated in Figure 2 (middle), the Spatial Enhancement Branch (SpaBlock) boosts spatial representations through an exactly invertible and nonlinear transformation. The input feature map *X* is channel-wise divided into $X_{1}$ and $X_{2}$ and then processed by a series of affine coupling layers:

(8) $$\begin{array}{cc} \left[X_{1},X_{2}\right] & =\mathrm{split}(X),\\ Y_{1} & =X_{1}+\mathrm{RHB}\left(X_{2}\right),\\ S & =c\left(2\mathrm{Sigmoid}(\mathrm{RHB}\left(Y_{1}\right))-1\right),\\ Y_{2} & =X_{2}⊙\exp\left(S\right)+\mathrm{RHB}\left(Y_{1}\right),\\ \mathrm{Spa}\_\mathrm{out} & =\mathrm{Concat}(Y_{1},Y_{2}), \end{array}$$

where RHB (ResHINBlock, see Figure 2, right) serves as the nonlinear mapping function. The combined additive and multiplicative couplings jointly preserve all information while enhancing the expressive power of the features.

The invertible affine coupling in SpaBlock is designed to preserve fine spatial gradients during nonlinear enhancement. In low-light conditions, high-frequency spatial details such as fine textures and edges are easily degraded. Preserving these components during nonlinear transformation is essential for downstream tasks like illumination prior estimation. Standard convolutions tend to smooth or alias these components. The coupling mechanism avoids this by ensuring mutual information flow between feature partitions—transformations applied to one partition are conditioned on the other, preventing irreversible information collapse.

RHB stabilizes feature learning via Half-Instance Normalization (HIN): by normalizing only half the channels, it preserves native statistics in one branch while regularizing the other—preventing over-smoothing and enhancing sensitivity to local contrast, which is critical under low-light degradation.

The dual-path design with residual connections ensures gradient stability during training, enabling deep feature refinement without vanishing activations.

The ResHINBlock (RHB) introduces Half-Instance Normalization (HIN), preserving partial feature statistics while normalizing the rest to stabilize training and improve generalization. Given input feature map *F*, HIN splits it channel-wise into $F_{a}$ and $F_{b}$, applies Instance Normalization (IN) to $F_{a}$, and concatenates $\mathrm{IN}(F_{a})$ with $F_{b}$.

(9) $$\mathrm{HIN}\left(F\right)=\mathrm{Concat}\left(\mathrm{IN}(\mathrm{Split}(F)),\;\mathrm{Split}(F)\right).$$

Using this operator, the forward path of RHB, consisting of a main branch with two 3×3 convolutions and a residual 1×1 convolution, is expressed as

(10) $$\mathrm{RHB}\left(x\right)=\mathrm{LReLU}\;\left({\mathrm{Conv}}_{3\times 3}\left(\mathrm{LReLU}\left(\mathrm{HIN}\left({\mathrm{Conv}}_{3\times 3}\left(x\right)\right)\right)\right)\right)+{\mathrm{Conv}}_{1\times 1}\left(x\right).$$

### 3.2. Dual-Branch Spectral-Spatial Attention

#### 3.2.1. Frequency-Modulated Attention Block

The Frequency-Modulated Attention Block (FMAB) is designed to model global illumination relationships—a task fundamentally misaligned with the local receptive field of spatial-domain operators. In low-light images, degradation manifests as non-uniform energy suppression across the spatial domain, but its root cause lies in the frequency domain: photon deficiency compresses the amplitude spectrum, reducing dynamic range and flattening contrast. Spatial attention, which computes dot products between local patches, can only redistribute features within limited neighborhoods—it cannot recover globally suppressed energy patterns.

FMAB addresses this by shifting the attention computation to the Fourier domain. Here, illumination structure is compactly represented as low-frequency amplitude distributions, and long-range brightness dependencies become local operations in spectral space. The conjugate product $F_{q}⊙\mathrm{conj}\left(F_{k}\right)$ computes the cross-power spectrum—the Fourier dual of spatial cross-correlation. This operation measures how well the global energy profiles of *q* and *k* align, producing an attention map that emphasizes regions with coherent illumination levels across the entire image. Unlike spatial attention—which matches local texture patterns—FMAB matches global energy distributions, making it inherently suited for illumination balancing.

Critically, FMAB integrates the external amplitude prior $|F_{\mathrm{mod}}|$ from MSAE to guide this process. Rather than learning attention weights purely from data, FMAB uses physically meaningful priors to modulate feature responses—transforming attention from a generic similarity measure into a targeted energy compensation operator.

**Figure 3:**
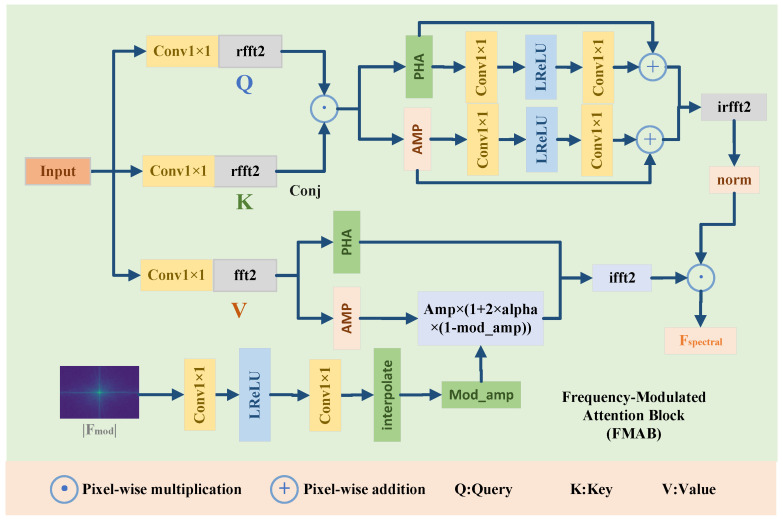
The framework of the proposed FMAB.

In the FMAB (see Figure 3), we first use a 1×1 convolution to obtain the query *q*, key *k*, and value *v*:

(11) $$q,k,v=\mathrm{Split}({\mathrm{Conv}}_{1\times 1}\left(x\right)),$$

We then feed q and k into the FFT and compute their conjugate product to form the frequency-domain attention:

(12) $$\begin{array}{c} {\mathrm{Attn}}_{\mathrm{freq}}=rfft2\left(q\right)⊙\mathrm{conj}\left(rfft2\left(k\right)\right), \end{array}$$

This spectrum is refined by SpecRefine (a dual-branch convolution on magnitude and phase), followed by a 2D inverse real FFT and LayerNorm to obtain the global attention map:

(13) $${\mathrm{Attn}}_{\mathrm{map}}=\mathrm{LN}\left(irfft2\left(\mathrm{SpecRefine}\left({\mathrm{Attn}}_{\mathrm{freq}}\right)\right)\right),$$

where LN(·) denotes Layer Normalization with learnable bias. The external prior magnitude $\left|F_{\mathrm{mod}}\right|$ is adapted using a lightweight remapper consisting of Conv_1×1_, LReLU and Conv_1×1_, and is aligned via interpolation.

(14) $${\mathrm{Mod}}_{\mathrm{amp}}=\mathrm{Interpolate}\left({\mathrm{Conv}}_{1\times 1}\left(\mathrm{LReLU}\left({\mathrm{Conv}}_{1\times 1}\left(\left|F_{\mathrm{mod}}\right|\right)\right)\right)\right).$$

The value tensor is modulated in the frequency domain by adjusting its magnitude and then recovered via ifft2:

(15) $$\begin{array}{cc} \left|V\right| & =\mathrm{abs}(fft2(v)),\\ \left|V_{\mathrm{mod}}\right| & =\left|V\right|⊙\left[1+2\alpha (1-{\mathrm{Mod}}_{\mathrm{amp}})\right],\\ V_{\mathrm{mod}} & =ifft2\left(\left|V_{\mathrm{mod}}\right|\;e^{j∠V}\right), \end{array}$$

where α is a learnable scalar. In low-light enhancement, the primary objective is to recover visibility by compensating for luminance deficiency—a task fundamentally governed by energy (amplitude) redistribution. Structural fidelity, while important, is primarily handled by spatial-domain branches (e.g., SVDAB) that model local textures and edges. Modulating phase in FMAB would interfere with this functional separation: since phase encodes geometric layout, altering it introduces unnecessary risk of structural distortion without contributing to the core goal of illumination recovery. Therefore, amplitude-only modulation is not only sufficient but preferable—it focuses FMAB on its designated role (global energy balancing) while leaving structural refinement to specialized spatial modules.

This modulation step performs explicit information compensation, where the factor $\left[1+2\alpha \left(1-{\mathrm{Mod}}_{\mathrm{amp}}\right)\right]$ adaptively boosts the signal magnitude, applying greater amplification to channels corresponding to higher information loss (i.e., low ${\mathrm{Mod}}_{\mathrm{amp}}$ values). Concurrently, preserving the original phase ∠V ensures that this energy restoration process maintains the image’s structural integrity.

(16) $$F_{\mathrm{spectral}}=V_{\mathrm{mod}}⊙{\mathrm{Attn}}_{\mathrm{map}}.$$

#### 3.2.2. Scale-Variant Depth Attention Block

**Figure 4:**
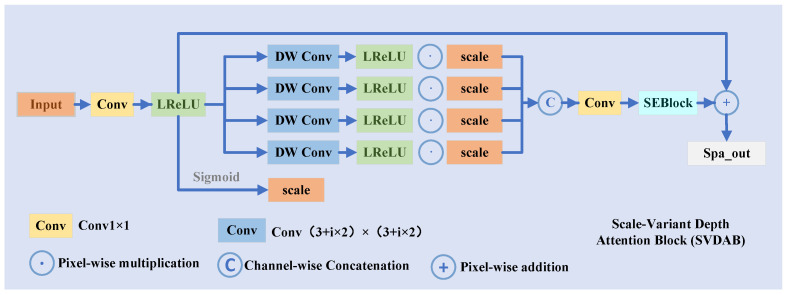
The framework of the proposed SVDAB.

As shown in Figure 4, we propose MultiConvBlock to extract multi-scale features through depthwise separable convolutions with varying kernel sizes and attention gating.

The Scale-Variant Depth Attention Block (SVDAB) is designed to complement the global, frequency-guided enhancement of FMAB with fine-grained spatial refinement. While FMAB recovers illumination by modulating spectral energy based on MSAE’s prior, it operates at a global level and lacks sensitivity to local structural variations—such as textures, edges, and fine gradients—which are critical for perceptual quality.

To address this, SVDAB employs multi-scale depthwise convolutions with adaptive kernel sizes ($k_{i}=3+2i$) to simultaneously capture features across different spatial granularities—from fine details (small kernels) to broader contextual structures (large kernels). Crucially, all branches share a lightweight gating signal *s*, derived from the input feature, enabling content-aware activation: regions requiring detail recovery (e.g., texture-rich or edge-heavy areas) are selectively enhanced, while smooth regions are preserved to avoid noise amplification.

This design differs fundamentally from conventional multi-scale modules (e.g., ASPP, Inception) in two ways: (1) depthwise separable convolutions ensure computational efficiency while preserving spatial resolution; (2) the shared gating mechanism enforces cross-scale feature consistency, preventing conflicting responses across scales. The fused features are further recalibrated via an SEBlock to emphasize informative channels.

Underlying this design is a *divide-and-conquer* philosophy: by decoupling global illumination modeling (FMAB) from local structure refinement (SVDAB), we enable each branch to specialize in its respective domain—leading to spatially adaptive, structurally coherent enhancements that synergize with, rather than compete against, FMAB’s global correction. The input *x* is reduced and activated:

(17) $$\begin{array}{cc} f_{\mathrm{shared}} & =\mathrm{LReLU}({\mathrm{Conv}}_{1\times 1}\left(x\right)),\\ s & =\mathrm{sigmoid}(f_{\mathrm{shared}}). \end{array}$$

Each of the N=4 depthwise branches processes $f_{\mathrm{shared}}$ using different kernel size $k_{i}=3+2i$, followed by gating:

(18) $$F_{i}=\mathrm{LReLU}\left({\mathrm{DWConv}}_{i}\left(f_{\mathrm{shared}}\right)\right)⊙s.$$

The fused and recalibrated output is

(19) $$\begin{array}{cc} F_{\mathrm{fused}} & ={\mathrm{Conv}}_{1\times 1}\left(\mathrm{Concat}\left(F_{0},\ldots ,F_{N-1}\right)\right),\\ F_{\mathrm{att}} & =\mathrm{SEBlock}(F_{\mathrm{fused}}). \end{array}$$

Final spatial-enhanced feature is computed as

(20) $$F_{\mathrm{spatial}}=x+F_{\mathrm{att}}.$$

### 3.3. Spectral Gated Feed-Forward NetworK

To model both spatial and frequency representations, we propose the Spectral Gated Feed-Forward Network (SGFFN). It performs learnable frequency filtering within local patch regions.

Standard feed-forward networks operate purely in the spatial domain, treating all frequency components implicitly—often leading to suboptimal enhancement of energy-suppressed bands or unintended amplification of noise. Global frequency-domain methods, while effective for illumination adjustment, tend to lose fine spatial localization when applied naively. SGFFN addresses this gap by introducing localized spectral processing: features are partitioned into non-overlapping patches before undergoing Fourier transform, allowing frequency modulation to remain spatially adaptive. This local patch-wise strategy prevents the blurring and ringing artifacts commonly associated with global spectral operations, while preserving structural coherence at boundaries.

Instead of relying on fixed or handcrafted filters as in prior frequency-aware designs, SGFFN employs a learnable complex-valued kernel $W_{\mathrm{fft}}$ that adapts dynamically to the spectral characteristics of each patch. This enables content-aware enhancement—boosting frequencies where information is deficient, while suppressing bands dominated by noise or aliasing. Following inverse transformation, a dual-path gating mechanism further refines the features: the output of a 3×3 convolution is split into two branches, each modulated by the GELU-activated counterpart. This cross-gating interaction encourages selective feature activation, enhancing discriminative frequencies while attenuating irrelevant or corrupted components—a behavior not achievable with conventional linear or ReLU-based FFNs.

Positioned after the attention modules, SGFFN serves as a frequency-aware refinement stage that complements the global guidance from FMAB and the local modeling from SVDAB. Rather than repeating their functions, it operates at an intermediate scale—selectively sharpening spectral details that earlier stages may have coarsely adjusted, thereby closing the loop between frequency prior estimation and spatial reconstruction. Given the input $x\in R^{B\times C\times H\times W}$, we first expand it into $x_{\exp}$, and partition it into non-overlapping patches $\left\{P_{i}\right\}$ of size p×p:

(21) $$F_{\mathrm{filtered}}\left(P_{i}\right)=rfft2\left(P_{i}\right)⊙W_{\mathrm{fft}},$$

where $W_{\mathrm{fft}}$ is a learnable spectral filter applied in the Fourier domain. It enables adaptive frequency-band enhancement by selectively amplifying or suppressing specific spectral components. Patches are inverse transformed via irfft(·) and reassembled:

(22) $$x_{\mathrm{filtered}}=\mathrm{RearrangeFromPatches}\left(\mathrm{irfft}\left(F_{\mathrm{filtered}}\left(P_{i}\right)\right)\right).$$

A convolution is applied, then the output is split and gated as

(23) $$\begin{array}{cc} x_{1},x_{2} & =\mathrm{Split}({\mathrm{Conv}}_{3\times 3}\left(x_{\mathrm{filtered}}\right)),\\ x_{\mathrm{fused}} & =\mathrm{GELU}\left(x_{1}\right)⊙x_{2}+\mathrm{GELU}\left(x_{2}\right)⊙x_{1}. \end{array}$$

Final output is computed via 1×1 projection:

(24) $$y={\mathrm{Conv}}_{1\times 1}\left(x_{\mathrm{fused}}\right).$$

### 3.4. Loss Function

To facilitate end-to-end training of the proposed AFMNet, we define a composite loss function $L_{\mathrm{total}}$, which integrates constraints at the pixel, perceptual, structural, and frequency levels:

(25) $$L_{\mathrm{total}}=\lambda_{\mathrm{pix}}L_{\mathrm{pix}}+\lambda_{\mathrm{vgg}}L_{\mathrm{vgg}}+\lambda_{\mathrm{ssim}}L_{\mathrm{ssim}}+\lambda_{\mathrm{freq}}L_{\mathrm{freq}}.$$

The pixel-wise loss uses an adaptive brightness-based weight:

(26) $$\begin{array}{cc} w & =\exp(-\gamma \cdot \mathrm{Mean}\left(I_{\mathrm{GT}}\right)),\\ L_{\mathrm{pix}} & =\mathrm{Mean}(w\cdot |I_{\mathrm{enh}}-I_{\mathrm{GT}}|), \end{array}$$

where $I_{\mathrm{enh}}$ and $I_{\mathrm{GT}}$ denote the enhanced image and ground-truth image, respectively, and γ=3.0 adjusts sensitivity. To capture perceptual quality, we define a VGG-based L1 loss:

(27) $$L_{\mathrm{vgg}}={∥\Phi_{i}\left(I_{\mathrm{enh}}\right)-\Phi_{i}\left(I_{\mathrm{GT}}\right)∥}_{1},$$

where $\Phi_{i}\left(\cdot \right)$ indicates features from the *i*-th layer of VGG-19. Structural details are preserved via SSIM loss:

(28) $$L_{\mathrm{ssim}}=1-\mathrm{SSIM}\left(I_{\mathrm{enh}},I_{\mathrm{GT}}\right).$$

The frequency-domain structural loss, $L_{\mathrm{freq}}$, is a core component of AFMNet designed to supervise the network in learning the correct spectral information distribution pattern. Our assumption is that a well-enhanced image should replicate the ground-truth’s pattern, not just its absolute energy. To this end, we adopt Cosine Similarity, which is invariant to absolute magnitude, to measure the structural similarity between the modulated spectrum $|F_{\mathrm{mod}}|$ and the ground-truth spectrum $|F_{\mathrm{GT}}|$. This loss encourages the MSAE to learn a physically meaningful spectral energy distribution, effectively teaching the network how information should be organized in the frequency domain. It is formulated as

(29) $$L_{\mathrm{freq}}=1-\mathrm{Mean}(\mathrm{Cos}\mathrm{ineSimilarity}(|F_{\mathrm{mod}}\left|,\right|F_{\mathrm{GT}}|)),$$

where $|F_{\mathrm{mod}}|$ (refer to Equation (5)) is the modulated amplitude spectrum output by the MSAE module. This loss encourages the two spectra to align in direction, guiding the MSAE to learn a physically meaningful spectral energy distribution, which provides a strong structural prior for subsequent spatial-domain refinement.

## 4. Experiments and Analysis

### 4.1. Implementation Details

The AFMNet model was implemented in PyTorch 2.1.1 and trained for $2\times 10^{5}$ iterations using the Adam optimizer, with parameters set to $\beta_{1}=0.9$ and $\beta_{2}=0.999$. The initial learning rate was $4\times 10^{-4}$, which was subsequently annealed to $1\times 10^{-7}$ following a cosine annealing schedule. The model was trained on 128×128 pixel patches randomly cropped from low- and normal-light image pairs, with a batch size of 8. For data augmentation, we applied random rotations and horizontal flips to the training samples.

### 4.2. Datesets and Metric

We evaluated our method on several public low-light enhancement datasets, including LOL (v2-real [9], and v2-synthetic [9]) and SDSD-indoor [10]. LOLv2-real contains 689 training and 100 testing pairs. LOLv2-synthetic is synthetically generated from RAW image data. For SDSD-indoor, we use the static indoor subset, captured using a Canon EOS 6D Mark II (Canon Inc., Tokyo, Japan) with an ND filter. This subset provides 62 training and 6 testing video pairs.

### 4.3. Compared Methods

To evaluate the performance of our method, we conducted a comprehensive comparison of the proposed AFMNet against various state-of-the-art deep learning methods, including Kind [4], MIRNet [22], RetinexDIP [23], KinD++ [24], RUAS [6], SNR-Aware [12], URetinex [25], CLIP-LIT [26], FourLLIE [19], GSAD [27], NeRCo [28], Retinexformer [17], AnlightenDiff [18], Pseudo [29], CSPN [30], MPC-Net [31], DMFourLLIE [20], IGDFormer [32], DiffDark [33], and KANformer [34].

These methods were trained on the same low-light image enhancement datasets using their officially provided public code (Supplementary Materials). We used three mainstream image quality assessment metrics for objective evaluation: Peak Signal-to-Noise Ratio (PSNR), Structural Similarity (SSIM), and the Learned Perceptual Image Patch Similarity (LPIPS). Higher PSNR and SSIM values, along with a lower LPIPS value, represent a better enhancement effect.

### 4.4. Visual Comparisons

#### 4.4.1. Quantitative Analysis

As summarized in Table 1, AFMNet is compared with state-of-the-art methods on the LOL-v2-real and LOL-v2-synthetic subsets. AFMNet delivers the highest PSNR values of 23.15 dB and 25.97 dB, respectively, and simultaneously attains the best SSIM scores (0.868/0.942), revealing superior luminance and structure restoration. Regarding perceptual quality, it ranks second on LOL-v2-real (LPIPS = 0.039) and first on LOL-v2-syn (LPIPS = 0.023). Despite these gains, the network requires only 14.52 G FLOPs and 4.18 M parameters, indicating a favorable performance–efficiency trade-off. These results verify the strong generalization ability of AFMNet in both real and synthetic low-light scenarios.

Table 2 presents a quantitative comparison of enhancement performance on the SDSD-indoor subset. The SDSD dataset consists of real-world video frames with exhibiting diverse textures, dynamic objects, and drastic illumination changes, which pose a significant challenge for image enhancement models in terms of robustness and fine-grained detail recovery. On the indoor subset, AFMNet achieves a PSNR of 31.17 dB and an SSIM of 0.900, outperforming all other competing methods. It surpasses models such as SNR-Aware, MambaIR, and Mamballie in terms of PSNR, while achieving the highest SSIM among all evaluated approaches.

To evaluate the generalization performance of our model, we conduct a quantitative comparison with eight state-of-the-art methods on four unpaired datasets: LIME, DICM, NPE, and MEF, as shown in Table 3. We adopt the NIQE metric for evaluation, where lower scores indicate better perceptual quality. AFMNet achieves the best performance on both the NPE and MEF datasets, with NIQE scores of 3.88 and 3.49, respectively, substantially outperforming all other methods. On the LIME and DICM datasets, AFMNet also obtains competitive results, closely approaching the top scores. These results demonstrate the strong generalization and robustness of AFMNet in diverse low-light imaging conditions, even without paired supervision.

**Table 1:** Quantitative comparison with state-of-the-art methods. Best results are in **bold**, second-best are underlined.

Methods	Venue	LOL-v2-real			LOL-v2-syn			Complexity	
		PSNR (dB)↑	SSIM↑	LPIPS↓	PSNR (dB)↑	SSIM↑	LPIPS↓	FLOPs (G)↓	Params (M)↓
Kind [4]	ACMM’19	17.54	0.669	0.375	16.25	0.591	0.435	34.99	8.02
MIRNet [22]	ECCV’20	22.11	0.794	0.145	22.52	0.900	0.057	785	31.76
RetinexDIP [23]	TCSVT’21	14.68	0.495	0.348	15.91	0.762	0.214	-	-
KinD++ [24]	IJCV’21	20.01	0.841	0.143	22.62	0.904	0.067	7484.32	8.28
RUAS [6]	CVPR’21	18.37	0.723	-	16.55	0.652	-	0.83	0.003
SNR-Aware [12]	CVPR’22	21.48	0.849	0.169	24.14	0.928	0.056	26.35	4.01
URetinex [25]	CVPR’22	21.16	0.840	0.144	24.14	0.928	-	5.61	0.08
CLIP-LIT [26]	ICCV’23	15.18	0.529	0.369	16.19	0.775	0.204	30.58	51.84
FourLLIE [19]	ACMM’23	22.34	0.847	0.159	24.65	0.919	0.066	13.4	0.12
GSAD [27]	NeurIPS’23	20.15	0.845	0.113	24.47	0.928	0.063	29.58	23.32
NeRCo [28]	ICCV’23	19.45	0.733	0.300	20.87	0.815	0.148	21.04	8.44
Retinexformer [17]	ICCV’23	22.43	0.811	0.249	25.627	0.931	0.079	17.02	1.61
AnlightenDiff [18]	TIP’24	20.65	0.837	0.146	-	-	-	23.97	13.91
Pseudo [29]	TCSVT’24	18.46	0.81	0.153	17.88	0.76	0.142	1.48	1.54
CSPN [30]	TCSVT’24	21.59	0.859	0.0917	-	-	-	60.92	1.40
MPC-Net [31]	TCSVT’24	22.60	0.864	0.103	25.67	0.939	0.044	4.4	-
DMFourllie [20]	ACMM’24	22.64	0.858	0.052	25.83	0.931	**0.023**	1.69	0.41
IGDFormer [32]	PR’25	22.73	0.833	-	25.33	0.937	-	9.68	3.55
DiffDark [33]	PR’25	20.84	0.868	0.1037	24.08	0.915	0.095	-	-
KANfourmer [34]	ACMM’25	21.851	0.852	**0.034**	25.56	0.932	0.085	6.221	1.88
AFMNet (Ours)	-	**23.15**	**0.868**	0.039	**25.97**	**0.942**	**0.023**	14.519	4.176

**Table 2:** Quantitative comparison on the SDSD-indoor [10] dataset in terms of PSNR and SSIM. The best results are in **bold**, and the second best are underlined.

Methods	PSNR (dB) ↑	SSIM ↑	Methods	PSNR (dB) ↑	SSIM ↑
Kind	21.95	0.672	Retinexformer	29.77	0.896
MIRNet	24.38	0.864	MambaIR	28.97	0.884
RUAS	23.17	0.696	ECAFormer	29.11	0.874
SDSD	25.20	0.722	QuadPrior	22.22	0.783
Retinex	23.17	0.696	Mamballie	30.12	0.900
Restormer	25.67	0.827	Ours	**31.17**	**0.900**
SNR-Aware	29.44	0.894			

**Table 3:** NIQE scores on LIME, DICM, NPE, and MEF datasets. The best results are in **bold** and the second-best are underlined. All evaluated methods have been pre-trained on the LOLv2-synthetic dataset.

Methods	LIME	DICM	NPE	MEF
Kind	4.772	3.614	4.175	4.819
MIRNet	6.453	4.042	5.235	5.504
SGM	5.451	4.733	5.208	5.754
FECNet	6.041	4.139	4.500	4.707
HDMNet	6.403	4.773	5.108	5.993
Bread	4.717	4.179	4.160	5.369
SNR	4.618	**3.227**	3.975	4.589
Retinexformer	**3.441**	4.008	3.893	3.727
FourLLIE	4.402	3.374	3.909	4.362
AFMNet	4.2	3.94	**3.88**	**3.49**

#### 4.4.2. Qualitative Analysis

**Figure 5:**
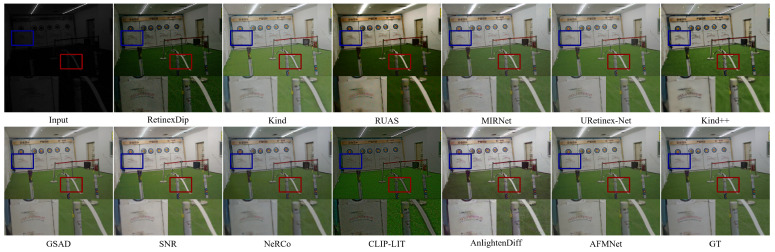
Visual comparison on LOL-v2-real.

As illustrated in Figure 5, the LOL-v2-real dataset poses considerable challenges for low-light enhancement. RetinexDip fails to brighten dark regions, and Kind/Kind++ cause overexposure and color distortion, especially on the quiver (red box). RUAS and GSAD reduce noise but blur important textures, such as the paper details in the blue box. MIRNet and URetinex-Net tend to over-smooth or leave residual darkness, while Mamballie introduces color inconsistencies despite relatively high brightness. In contrast, AFMNet produces visually natural results with balanced brightness, restored textures, and better structural fidelity across both marked regions. These results highlight AFMNet’s robustness and effectiveness in complex real-world low-light conditions.

**Figure 6:**
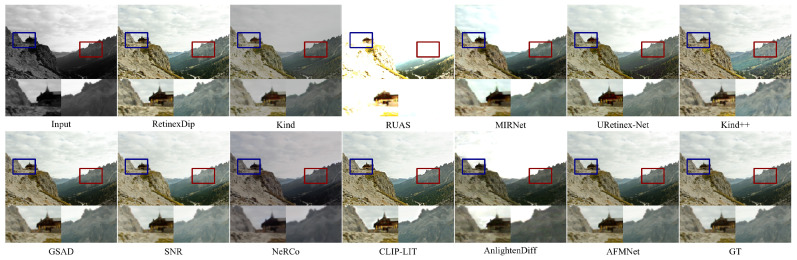
Visual comparison on LOL-v2-synthetic.

On the LOL-v2-synthetic set Figure 6, the low-contrast input poses a significant challenge. Existing methods often either induce overexposure, losing rock strata details in the red box (Kind++, RUAS), or over-smooth the image, distorting the temple’s decorative lines in the blue box (MIRNet, GSAD). Others flatten the contrast, blurring the temple’s silhouette. In contrast, AFMNet achieves a precise illumination redistribution, rendering sharp edges and natural colors on the temple while restoring clear, stratified textures on the mountain ridge, free from significant noise or artifacts.

**Figure 7:**
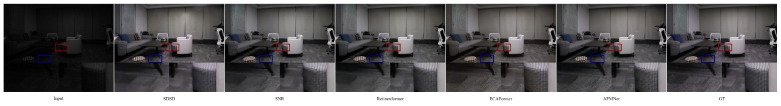
Visual comparison on SDSD.

As shown in Figure 7, the SDSD dataset presents severe low-light challenges. On the indoor subset (top row), competing methods tend to over-smooth textures, introduce color shifts on sofas, or fail to reveal shadow details. In contrast, AFMNet effectively preserves fine textures and restores accurate colors on both the floor and furniture, closely matching the ground truth.

**Figure 8:**
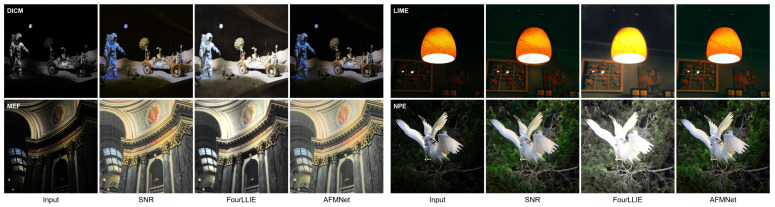
Visual comparison on four unpaired datasets (DICM, LIME, MEF, NPE).

To evaluate our model under unpaired conditions, we present qualitative comparisons on four datasets: DICM, LIME, MEF, and NPE, as shown in Figure 8. On DICM, SNR under-exposes with a strong blue cast, while FourLLIE over-brightens with noise and smoothing. In contrast, AFMNet maintains neutral tones and reveals fine textures on the rover and ground. On LIME, SNR leaves a greenish background and FourLLIE causes highlight clipping and color shift near the lamp. AFMNet suppresses color casts, preserves warm tones, and restores wall details with low noise. On MEF, SNR appears dim, and FourLLIE loses contrast and texture in bright regions. AFMNet enhances structural details, avoids halos, and produces clean lighting transitions. On NPE, SNR fails to brighten the bird, while FourLLIE desaturates colors. AFMNet retains detail in feathers, preserves natural saturation, and controls noise effectively. These results demonstrate AFMNet’s ability to preserve contrast, color fidelity, and fine textures across various unpaired low-light scenarios.

### 4.5. Ablation Study

To evaluate the effectiveness of each architectural component, we perform an ablation study on the LOL-v2-real dataset, as shown in Table 4. Starting from AFMNet’s full configuration, we progressively remove or simplify specific modules to assess their individual benefits. w/o Freq. Prior Guidance: Removing MSAE and all frequency-domain components in FMAB degrades the model into a standard spatial-domain attention module, causing the largest performance drop (PSNR: −1.03 dB), which confirms the fundamental value of our dual-domain design. w/o Multi-Scale in MSAE: Simplifying MSAE to a single-scale version reduces performance by 0.31 dB, indicating that multi-scale context is beneficial for accurate prior estimation. FMAB w/o Prior Guidance: Disabling prior guidance in FMAB (while retaining its frequency-domain attention structure) leads to a 0.57 dB drop, demonstrating that FMAB’s core contribution lies in its synergy with MSAE’s physically meaningful prior. DBSSA w/o Spatial: Removing the spatial branch in DBSSA results in a 0.39 dB degradation, validating that local spatial refinement is essential and cannot be fully replaced by global frequency modeling. SSFBlock w/o Freq. Branch: Eliminating the frequency branch in SSFBlock causes a 0.67 dB drop, confirming that explicit frequency-domain feature extraction is critical for effective prior generation. SGFFN to MLP: Replacing SGFFN’s frequency-filtered path with a plain MLP leads to a 0.24 dB decay, showing that frequency-aware enhancement in the feed-forward network provides measurable gains. Collectively, these results highlight that each component contributes distinct and indispensable value: MSAE generates adaptive priors, FMAB executes guided frequency modulation, SVDAB refines local structures, and SGFFN enables spectral-selective enhancement—together forming a coherent, principled architecture.

**Table 4:** Ablation study on the core components of our AFMNet, evaluated on the LOL-v2-real dataset. Our full model achieves the best performance, demonstrating the effectiveness of each proposed module and its internal design.

Metric	AFMNet (Full Model)	w/o Freq. Prior Guidance	w/o Multi-Scale in MSAE	FMAB w/o Prior Guidance	DBSSA w/o Spatial	SSFBlock w/o Freq. Branch	SGFFN to MLP
PSNR (dB) ↑	23.15	22.12	22.84	22.58	22.76	22.48	22.91
SSIM ↑	0.868	0.841	0.861	0.855	0.860	0.852	0.863

To assess the contribution of each component in our compound loss function, we conduct a cumulative ablation study on the LOL-v2-real dataset, as summarized in Table 5. Starting from a baseline that uses only pixel-wise loss ($L_{\mathrm{pix}}$), we progressively add perceptual loss ($L_{\mathrm{vgg}}$), structural similarity loss ($L_{\mathrm{ssim}}$), and frequency-domain loss ($L_{\mathrm{freq}}$). The baseline with only $L_{\mathrm{pix}}$ yields limited performance (22.34 dB PSNR), often resulting in texture blur and structural degradation. Adding $L_{\mathrm{vgg}}$ improves perceptual quality (+0.41 dB), while $L_{\mathrm{ssim}}$ further enhances structural fidelity. The full loss function with $L_{\mathrm{freq}}$ achieves the best performance, confirming its critical role in guiding frequency prior learning and suppressing spectral distortions. Together, these results validate the effectiveness of our composite loss design, with frequency-domain supervision playing a pivotal role.

## 5. Conclusions

In this work, we presented AFMNet, a dual-domain framework that reframes low-light image enhancement from an information-theoretic perspective. By designing a Multi-Scale Amplitude Estimator (MSAE) to learn a content-aware information deficit prior, our model explicitly estimates where information is lost and guides the enhancement process through a novel frequency-modulated attention mechanism. This synergistic design, which couples prior estimation with guided reconstruction, achieves an excellent balance between non-uniform illumination correction and detail preservation, delivering state-of-the-art performance on multiple benchmarks. Our work demonstrates the power of integrating information-theoretic principles into network design, leading to a more interpretable and effective solution. While AFMNet is currently amplitude-centric, future work could explore explicit phase modeling. Moreover, the concept of frequency-prior guidance holds significant promise for other image restoration tasks like dehazing and deraining, where degradation characteristics are well-defined in the frequency domain.

## Figures and Tables

**Table 5:** Ablation study on the components of our compound loss function, evaluated on the LOL-v2-real dataset. The progressive addition of each component contributes to performance improvement.

Metric	Setting A	Setting B	Setting C	AFMNet (Ours)
$L_{pix}$	✓	✓	✓	✓
$L_{vgg}$		✓	✓	✓
$L_{ssim}$			✓	✓
$L_{freq}$				✓
PSNR (dB) ↑	22.34	22.75	22.98	23.15
SSIM ↑	0.849	0.858	0.864	0.868

## Data Availability

All datasets used in this study are publicly available and cited within the manuscript.

## Supplementary Materials

The following supporting information can be downloaded at: https://www.mdpi.com/article/10.3390/e27121220/s1.
